# Development of breast phantoms using a 3D printer and glandular dose evaluation

**DOI:** 10.1002/acm2.13408

**Published:** 2021-09-16

**Authors:** Dong‐Yeon Lee, Yong‐In Jo, Sung‐Hee Yang

**Affiliations:** ^1^ Department of Radiological Science College of Nursing Health Sciences and Human Ecology Dong‐Eui University Busan Republic of Korea; ^2^ Dongnam Institute of Radiological and Medical Sciences Busan Republic of Korea; ^3^ Department of Radiological Science College of Health Sciences Catholic University of Pusan Busan Republic of Korea

**Keywords:** 3D printer, breast phantom, glandular dose, glass dosimeter, simulation

## Abstract

In this study, breast phantoms were fabricated by emulating glandular and adipose tissues separately using a three‐dimensional (3D) printer. In addition, direct and quantitative glandular dose evaluations were performed. A quantitative method was developed to evaluate the glandular and adipose tissues separately when performing glandular dose evaluations. The variables used for glandular dose evaluation were breast thickness, glandular tissue ratio, and additional filter materials. The values obtained using a Monte Carlo simulation and those measured using a glass dosimeter were compared and analyzed. The analysis showed that as the glandular tissue ratio increased, the dose decreased by approximately 10%, which is not a significant variation. The comparison revealed that the simulated values of the glandular dose were approximately 15% higher than the measured values. The use of silver and rhodium filters resulted in a mean simulated dose of 1.00 mGy and 0.72 mGy, respectively, while the corresponding mean measured values were 0.89 mGy ± 0.03 mGy and 0.62 mGy ± 0.02 mGy. The mean glandular dose can be reliably evaluated by comparing the simulated and measured values.

## 1 INTRODUCTION

Recent developments in the medical sector have shown a tendency to integrate medical technology with Industry 4.0.^1^ Such integration includes spotlighted applications such as surgical robotics designed to minimize laparotomy, artificial intelligence‐based diagnosis and examination, and the manufacturing of medical devices using three‐dimensional (3D) printers.^2^, ^3^, ^4^ In particular, 3D printers are widely used in medical radiology for applications such as the development of auxiliary devices for imaging, creation of anthropomorphic models based on medical images, and production of phantoms to estimate radiation doses.^5^, ^6^, ^7^, ^8^

Mammography is a technique used for breast cancer screening in women.^9^, ^10^ It is performed by compressing the breast between two plates and using low‐dose X‐rays for breast imaging. Mammography is convenient in terms of cost and time, and above all, it can detect microcalcification lesions.^11^ However, this technique involves the use of a high tube current at low voltages, which increases the risk of radiation exposure. This issue is exacerbated by the fact that mammography requires multiple imaging from different angles.^12^, ^13^, ^14^ Therefore, it is important to quantitatively assess the radiation dose received by the breast during a screening mammogram.

The breast is typically composed of adipose and glandular tissues. To roughly describe its structure, glandular tissue spreads from the nipple toward the chest wall and is surrounded by adipose tissue.^15^ Since glandular tissue is more sensitive to radiation than adipose tissue, the glandular dose is considered to be the breast dose in a conservative dose assessment. Therefore, assessing the breast dose is tantamount to assessing the glandular dose.

Many studies have been conducted to assess the glandular dose. However, most of these studies do not distinguish between glandular and adipose tissues; instead, they have been treated as an intermingled mass when assessing the glandular dose.^16^, ^17^, ^18^ Consequently, these studies have a common limitation in that they assess the glandular dose only indirectly.

To overcome these limitations, the present study aims to perform direct and quantitative glandular dose evaluation by producing breast phantoms that emulate glandular and adipose tissues separately using 3D printing technology.

## 2 METHODS

**FIGURE 1:**
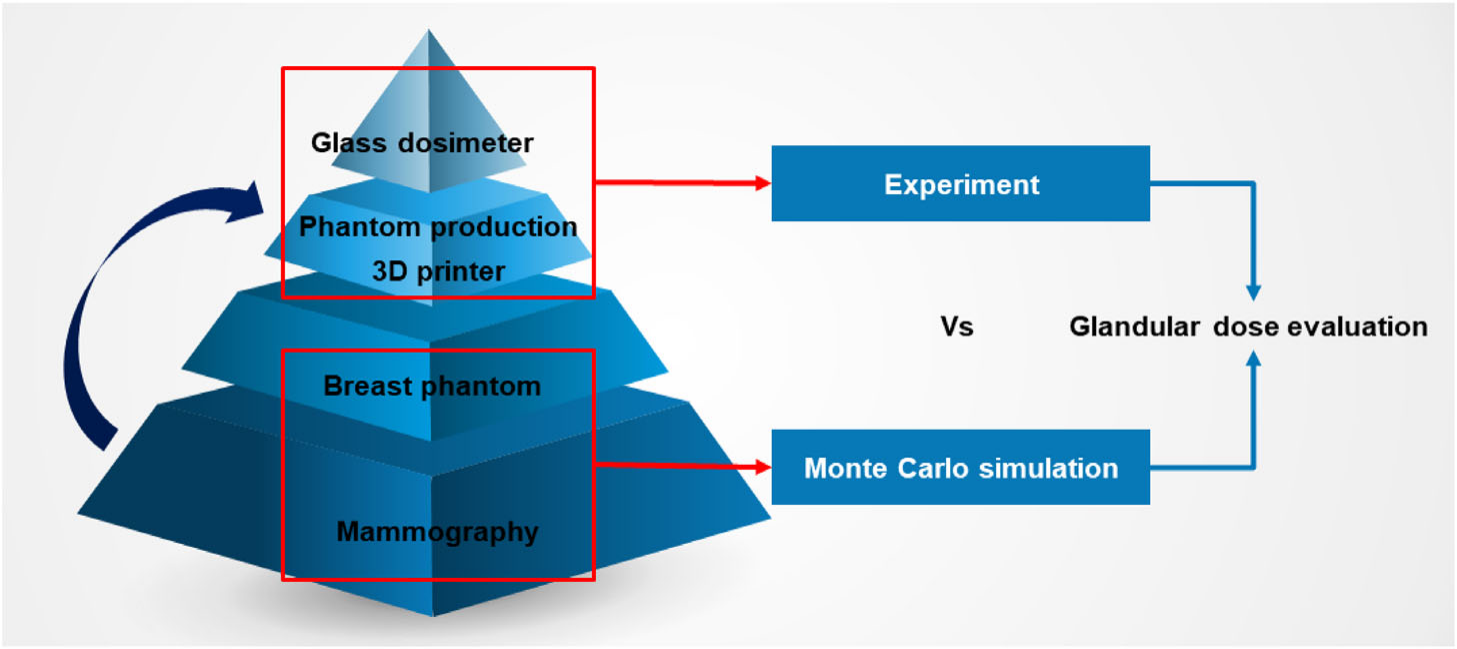
Overall procedural flow scheme followed in this study

For glandular dose evaluation, we compared and analyzed simulated values with those measured using a glass dosimeter on a breast phantom produced via 3D printing technology. Before producing the 3D‐printed breast phantom, we calculated the glandular dose by conducting a numerical study in virtual space using a Monte Carlo simulation (MCNPX Ver.2.7.0^19^), and the phantom was produced based on the simulated results. In general, simulated values are considered reliable when they show a relative error of less than 3%. Therefore, we set the number of iterations in the Monte Carlo simulation to 10^8^ and maintained the relative error of the resulting values within 2%. Figure 1 shows the overall procedural flow followed in this study.

### 2.1 Mammography equipment simulation

**FIGURE 2:**
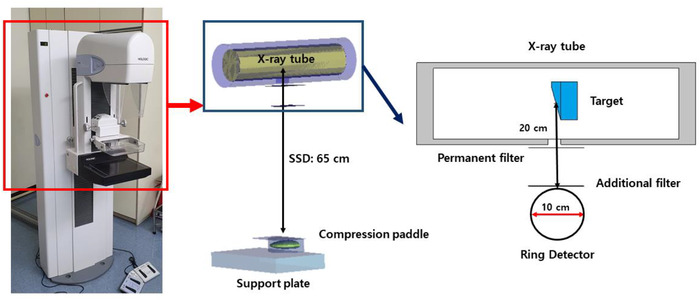
Mammography equipment simulated in this study

As a radiation source, we simulated the Selenia Full Field Digital Mammography System & Accessories (Hologic, Inc. Bedford, MA, USA), which is the equipment used in the Dongnam Institute of Radiological & Medical Sciences (DIRAMS). The overall architecture and geometry of the equipment are illustrated in Figure 2, and Table 1 presents the details of each component.

**FIGURE 3:**
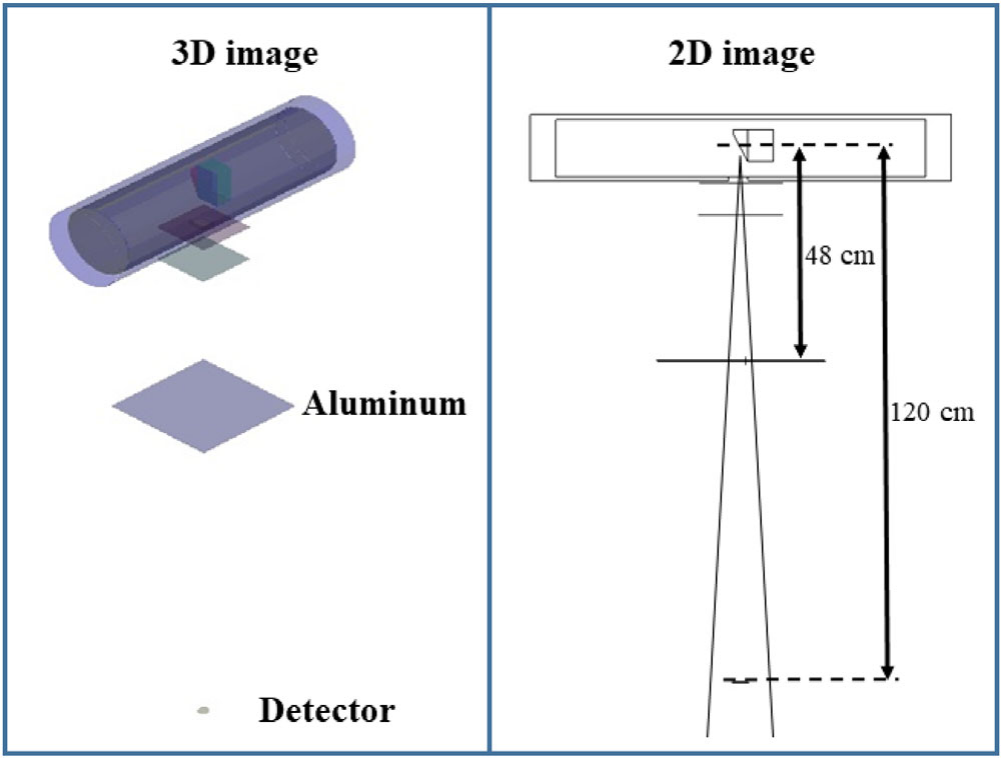
Geometry consideredfor the half‐value layer in the simulation^20^

**TABLE 1:** Composition and specification of the mammography equipment

Part	Material	Density (g/cm^3^)	Shape
Target	Tungsten (W, atomic no.: 74)	19.25	Target angle 10° Focal spot size: 0.3 mm
Permanent filter	Beryllium (Be, atomic no.: 4)	1.85	Thickness: 0.5 mm
Additional filter	Rhodium (Rh, atomic no.: 45)	12.41	Thickness: 0.06 mm
	Silver (Ag, atomic no.: 47)	10.49	Thickness: 0.06 mm
Compression paddle	Polycarbonate	1.2	Thickness: 2 mm
Support plate	Polycarbonate	1.2	Thickness: 5 cm

The most important aspect in the evaluation of the radiation dose is the simulation of the radiation source. Therefore, it is important to verify that the simulated mammography equipment in this study matches the structure of the actual equipment. The first factor to calculate for securing the reliability of the source was the quality of the radiation generated by the mammography equipment as a half‐value layer. The quality of the tube voltage of the mammography equipment used in DIRAMS was analyzed as 0.531 and 0.548 mm Al when the additional filter was rhodium and silver, respectively.^20^ We analyzed the X‐rays emitted by the simulated equipment to establish its reliability. After calculating the mean energy, spectral shape, and peak energy of the emitted X‐rays, as well as comparing the resulting values with those reported in the literature, the simulated equipment was determined to be reliable. For the X‐ray analysis, we set the tube voltage to 28 kVp and collected the emitted X‐rays in a virtual spherical detector. The detector was installed 20 cm below the target, and the spectrum was analyzed by dividing the collected X‐rays at 0.1 keV intervals.

### 2.2 Breast phantom simulation

**FIGURE 4:**
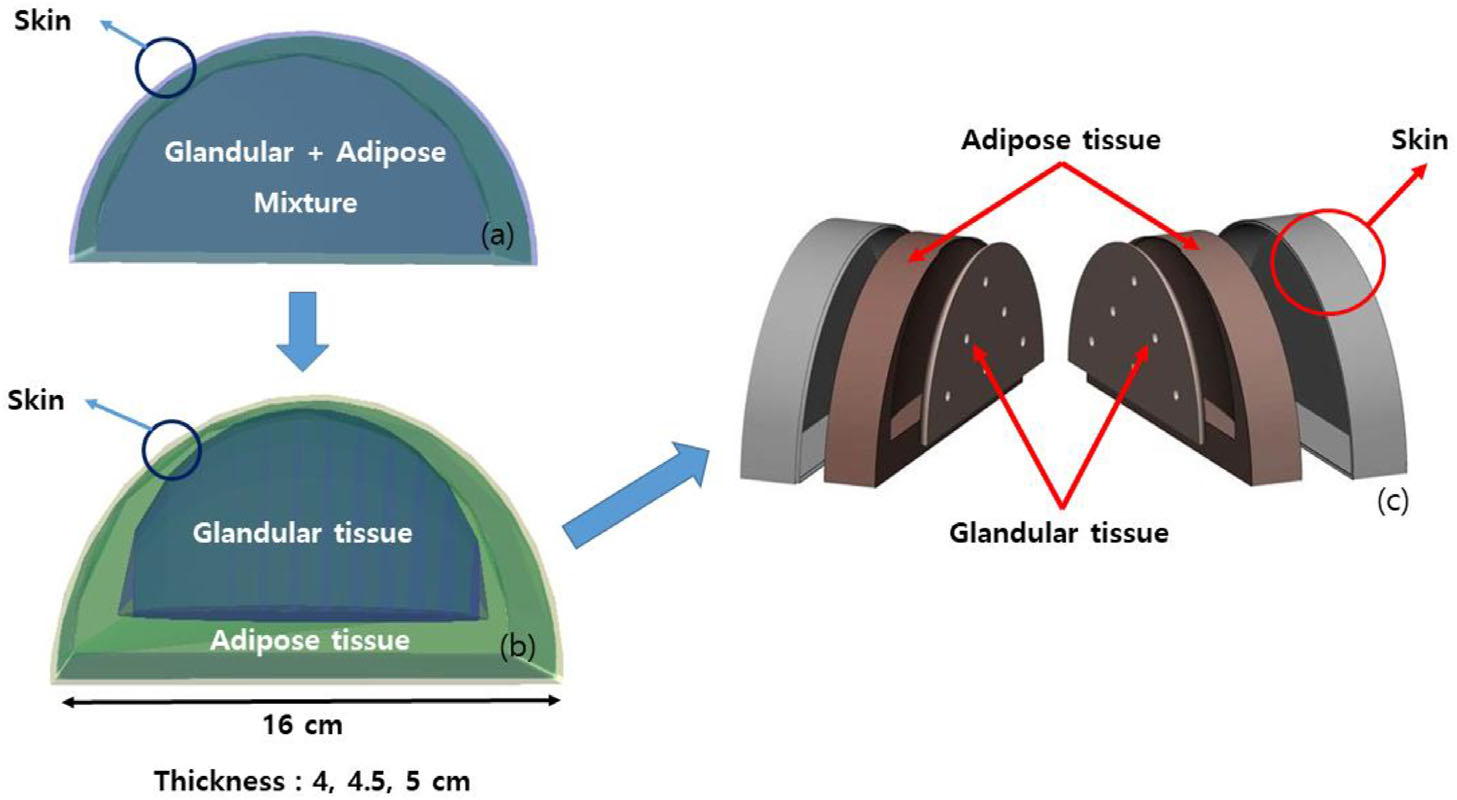
Shape and composition of the simulated breast phantom: (a) Mixture‐type breast phantom simulated in previous research. (b) Non‐mixture‐type breast phantom simulated in this study. (c) Composition of the non‐mixture‐type breast phantom

Previous studies on glandular dose evaluation did not distinguish between glandular and adipose tissues; instead, they considered these tissues as an intermingled mass. To avoid this shortcoming, we developed breast phantoms in which glandular and adipose tissues are simulated separately. Figure 4 shows the overall architecture and shape of the phantom simulated in this study. Specifically, as our basic concern is breast dosimetry for mammography, a compressed breast shape was simulated. Table 2 presents the chemical composition and density of the skin, glandular tissue, and adipose tissue, which are the major breast components.^21^ By referring to previous research on skin thickness according to body part,^22^ we set the skin thickness, that is, the outermost part of the breast, to 1.5 mm. The morphology of the mammary gland was simulated to have a spreading shape from the nipple toward the chest wall, and adipose tissue was simulated to fill the space not occupied by the glandular tissue. Breast sizes vary among individuals, as does the proportion of glandular tissue. Therefore, we produced a total of nine phantoms by setting the breast thickness to 4, 4.5, and 5 cm and applying glandular tissue ratios of 25%, 50%, and 75% for each thickness.

**TABLE 2:** Chemical composition and density of the breast components (skin, glandular tissue, and adipose tissue)

	Composition (%)		
Element	Skin	Glandular	Adipose
H	10	10.6	11.4
C	20.4	33.2	59.8
N	4.2	3	0.7
O	64.5	52.7	27.8
Na	0.2	0.1	0.1
P	0.1	0.1	–
S	0.2	0.2	0.1
Cl	0.3	0.1	0.1
Density (g/cm^3^)	1.09	1.02	0.95

### 2.3 Breast phantom production using 3D printing technology

**FIGURE 5:**
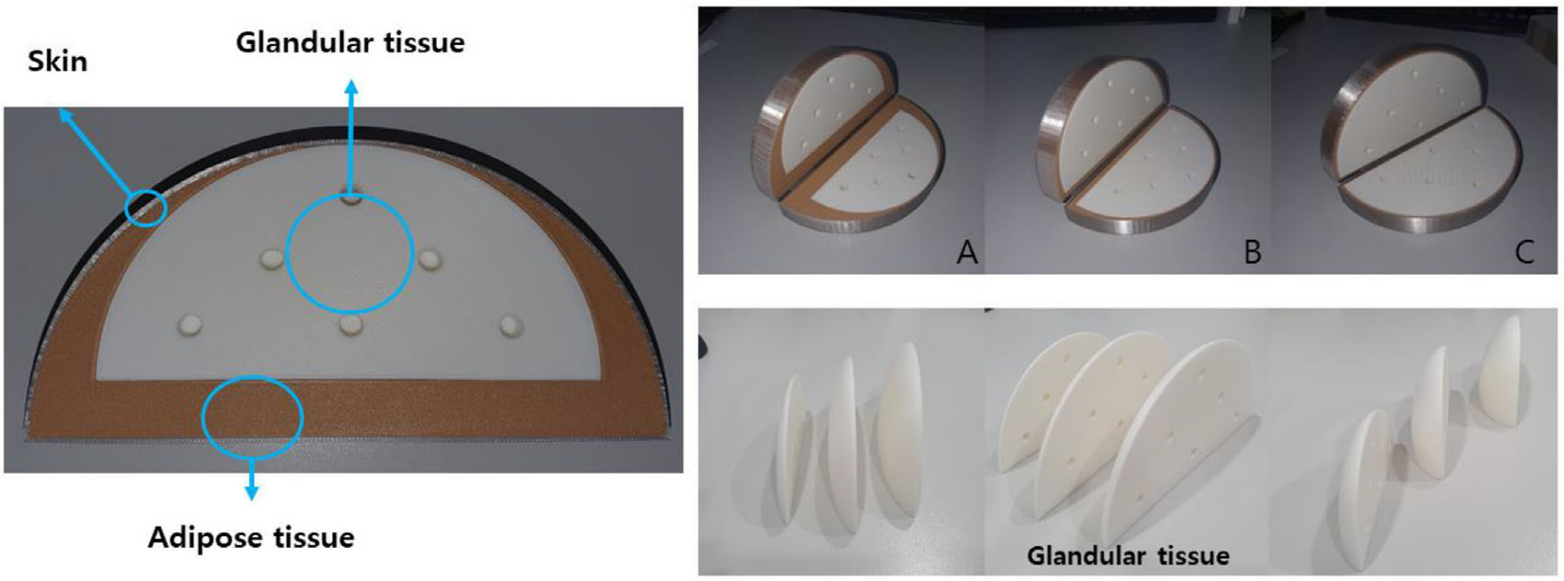
Architecture of the breast phantom produced using a 3D printer with different glandular tissue ratios (A = 25%, B = 50%, C = 75%) (right, top) and shapes (right, bottom)

Figure 5 illustrates the architecture of the simulated breast phantoms produced with a 3D printer. The printed phantoms have the same shape as the simulated one, with holes made within the glandular tissue portion to allow the insertion of glass dosimeters for direct glandular dose measurement. Six holes were made across the glandular tissue so that the entire portion is considered for glandular dose evaluation. The filament materials used for the production of the phantom were polycarbonate, polylactic acid, and WOOD for the skin, glandular tissue, and adipose tissue, respectively. The composition and density of the filament materials used for the breast phantoms are listed in Table 3. A study was conducted to determine which filament materials optimally resemble each body part,^23^ which in turn was based on a preliminary study on filament material selection for breast phantom production.^24^ Similar to the simulation, a total of nine phantoms were produced by combining three breast thickness (4, 4.5, and 5 cm) and three glandular tissue ratios (25%, 50%, and 75%).

**TABLE 3:** Composition and density of the filament materials used for producing the breast phantoms

Material	Density (g/cm^3^)	Composition (%)							
		C	O	Mg	Si	S	Cl	K	Ti
Polylacticacid (PLA)	1.25	54.76	44.99			0.13		0.12	
Polycarbonate (PC)	1.21	76.28	22.79			0.11			0.82
WOOD	1	61.48	37.65	0.26	0.47		0.15		

### 2.4 Glandular dose measurements using a glass dosimeter

**FIGURE 6:**
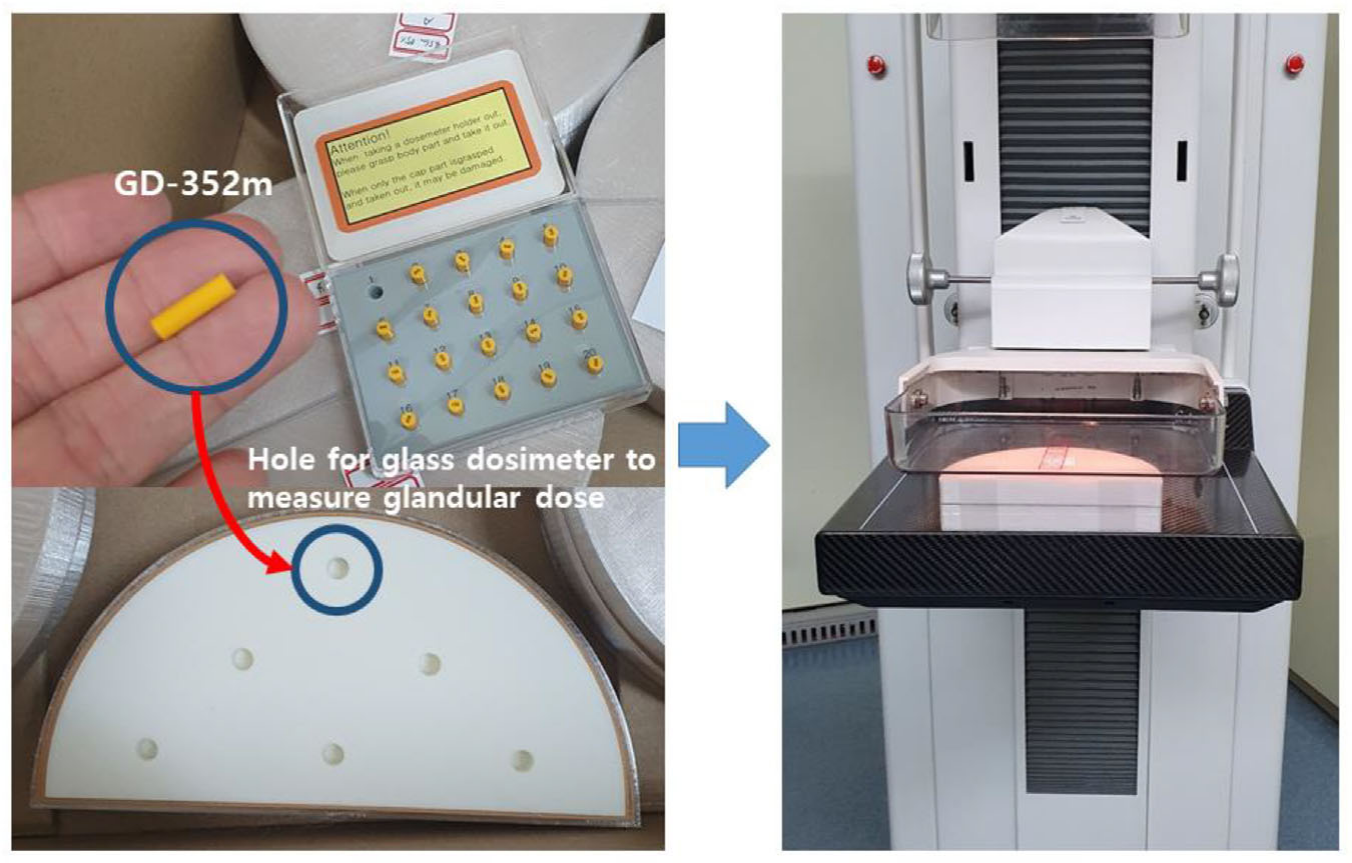
Glandular dose measurement using a glass dosimeter

The glandular dose of the 3D‐printed breast phantoms was measured using a glass dosimeter, which was installed in the portion of the phantoms occupied by glandular tissue, as shown in Figure 6. As a glass dosimeter filter, we used GD‐352 m, which is suitable for low‐energy ranges. FGD‐1000 (Asahi Techno Glass Co., Japan) was used as the reading system, and the average of 10 readings per dosimeter was taken as the resulting value. Before the actual measurements of the glandular dose of the 3D‐printed breast phantoms, the ambient background radiation was measured to exclude its contribution. Direct glandular dose measurements were made using the six glass dosimeters installed in the glandular tissue of each breast phantom. The mean value of the resulting measurements was taken as the final glandular dose of each breast phantom. The variability of the measured values was within 3%.

As previously mentioned, the mammography equipment model used in this study is the Selenia Mammography System, which is regularly calibrated and quality‐controlled by the Korea Institute of Medical Technology on a yearly basis. The last inspection and calibration took place on July 16, 2020. The imaging conditions for glandular dose evaluation were set as follows: a tube voltage of 28 kVp, a tube current of 55 mA, an irradiation field of 18 × 24 cm^2^, and a source–breast distance of 65 cm.

## 3 RESULTS

### 3.1 Photon analysis of mammography equipment

**FIGURE 7:**
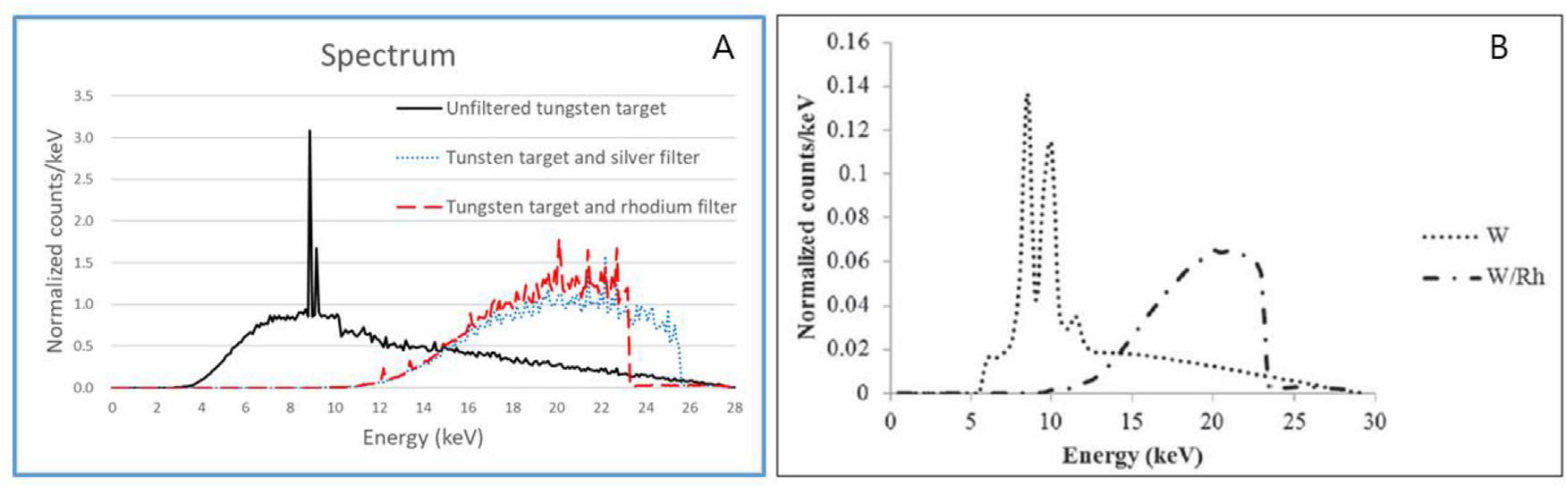
Spectral analysis of the X‐ray emitted from the X‐ray tube of the breast phantom simulated in this study ((a), calculated with MCNP) and in a previous study ((b)[24])

As shown in Figure 7, a spectral analysis was performed on the X‐rays emitted by the simulated mammography equipment to establish the reliability of the simulated radiation source.

The simulation‐calculated spectrum analysis revealed that when no additional filter was applied, the energy distribution increased to peak values at approximately 10–12 keV before decreasing. When additional filters were applied, the low‐energy area was attenuated, and high‐energy distributions appeared at approximately 18–22 keV. Peak energy values were observed at 8.9 and 9.2 keV when no additional filter was used.

### 3.2 Glandular dose evaluation according to breast thickness and glandular tissue ratio

**FIGURE 8:**
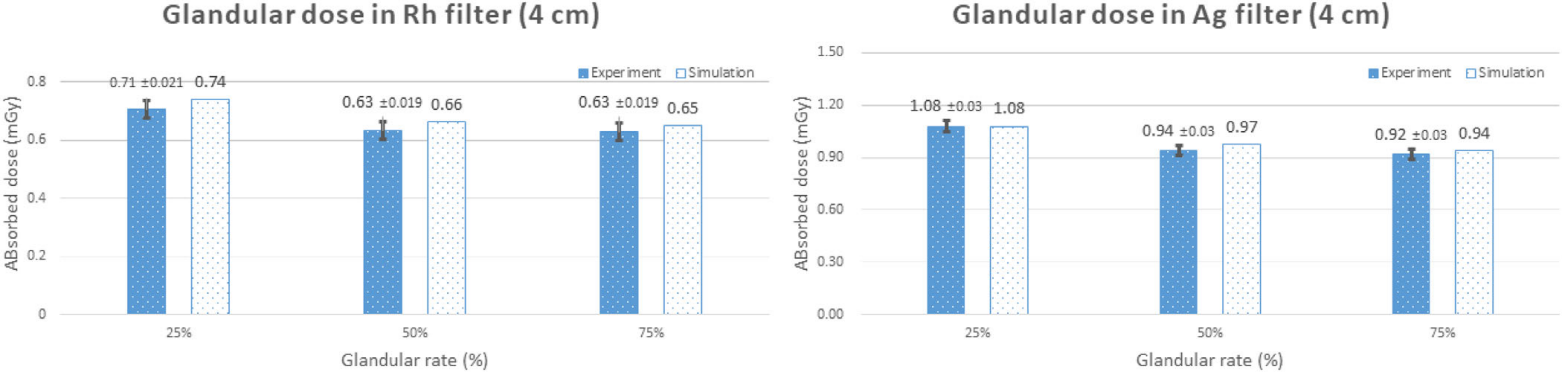
Comparison of the simulated and measured glandular dose values for a breast thickness of 4 cm

**FIGURE 9:**
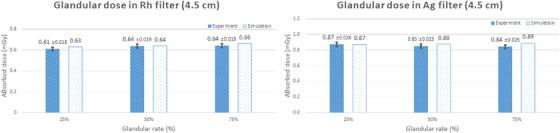
Comparison of the simulated and measured glandular dose values for a breast thickness of 4.5 cm

**FIGURE 10:**
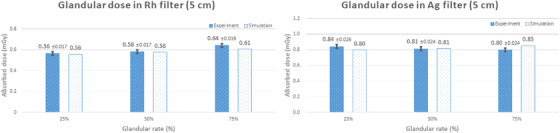
Comparison of the simulated and measured glandular dose values for a breast thickness of 5 cm

Three variables were taken into account for glandular dose evaluation: breast thickness, glandular tissue ratio, and additional filter type. Figures 8, 9, 10 present the comparison between the simulated and measured values according to the variation of these three variables.

The mean simulated values for a breast thickness of 4 cm when using the rhodium and silver filters were 0.68 mGy and 0.98 mGy, respectively. The mean measured values using the glass dosimeters were 0.66 mGy ± 0.02 mGy and 0.98 mGy ± 0.03 mGy, respectively. As for the dose variation with respect to the glandular tissue ratio, a higher glandular tissue ratio resulted in a lower dose.

The mean simulated values for a breast thickness of 4.5 cm when using the rhodium and silver filters were 0.64 mGy and 0.88 mGy, respectively. The mean measured values using the glass dosimeters were 0.62 mGy ± 0.02 mGy and 0.85 mGy ± 0.03 mGy, respectively. Regarding the dose variation with respect to the glandular tissue ratio, the simulated dose values increased slightly as the glandular tissue ratio increased, but no variation was observed in the measured values.

Finally, the mean simulated values for a breast thickness of 5 cm when using the rhodium and silver filters were 0.58 mGy and 0.82 mGy, respectively. The mean measured values using the glass dosimeters were 0.59 mGy ± 0.02 mGy and 0.83 mGy ± 0.02 mGy, respectively. Regarding the dose variation with respect to the glandular tissue ratio, the simulated and measured values behave in the same manner as for a breast thickness of 4.5 cm.

## 4 DISCUSSION

The results of this study can be summarized as follows. First, we analyzed the photon spectrum emitted by the simulated mammography equipment to verify the reliability of the values calculated by the simulation model. The results show that the shape and distribution of the overall spectrum were in good agreement with those reported in the literature.^25^, ^26^, ^27^, ^28^ In particular, high similarity was observed in the spectrum shape variation depending on the use of an additional filter. Consequently, the reliability of the simulated values obtained in this study was verified through the accuracy of the simulated mammography radiation.

Second, we compared the glandular dose values obtained in this study with those obtained in previous studies^11^, ^16^, ^17^, ^18^ to ensure their reliability. Notwithstanding the differences in numerical values due to different experimental conditions, such as the additional filter material and tube voltage, the discrepancies in the resulting values were within 5% of those found in previous studies conducted under similar conditions. Therefore, the reliability of the experimental values obtained in this study is established.

Third, the difference between the glandular dose values calculated by simulation and the values measured using a glass dosimeter was within approximately 5%. This may be due to the fact that the measured values are influenced by the surrounding environment, such as the temperature and humidity of the ambient, while these effects can be minimized in the simulation.

Fourth, glandular dose values were found to be 30%–40% higher with the silver filter than with the rhodium filter. This difference is attributed to the material and density of the additional filter, whereby a higher atomic number and density result in greater filtering of the X‐ray photons generated in the target. Although rhodium and silver have similar atomic numbers (45 and 47, respectively), rhodium has a significantly higher density than silver (12.41 g/cm^3^ vs. 10.49 g/cm^3^) and hence filters more photons. As a result, with a rhodium filter, less photons reach the glandular tissue, and therefore, the glandular dose is lower. The results of this study showed a similar trend to previous studies.^29^

Fifth, an analysis of the relationship between dose variation and glandular tissue ratio revealed that, contrary to the findings of previous studies,^16^, ^17^, ^18^ the glandular dose tends to decrease slightly as the glandular tissue ratio increases. This may be because the breast phantoms developed in this study separately simulated the glandular and adipose tissues, whereas previous studies did not distinguish them; instead, the previous studies treated them as an intermingled mass. More specifically, the absorbed dose is calculated as the absorbed energy per unit mass. In conventional mixture‐type breast phantoms, an increase in glandular tissue ratio leads to an increase in glandular dose because the glandular tissue mass is fixed. Conversely, the glandular dose does not increase in the non‐mixture‐type breast phantoms developed in this study, because the glandular tissue mass increases with an increase in the glandular tissue ratio. This result is consistent with that of a comparable study^30^ that assessed the glandular dose on a numerical voxel breast phantom, which further supports the reliability of the results of the present study.

## 5 CONCLUSIONS

We developed a breast phantom using 3D printing technology to enable quantitative glandular dose evaluation during mammography. This study differs from previous studies in that the glandular and adipose tissues were separately simulated when producing the breast phantom. Tissue separation allows for a direct calculation of the dose absorbed by glandular tissue, thus increasing the accuracy of the glandular dose evaluation. As a result, the glandular dose can be estimated more reliably with the non‐mixture‐type phantom developed in this study than with conventional mixture‐type phantoms. The significance of this study is reinforced by the fact that the reliability of the glandular dose estimated using the developed breast phantom was verified by comparing and analyzing the results obtained with a Monte Carlo simulation and the values measured using a glass dosimeter.

## AUTHOR CONTRIBUTIONS

Dong‐Yeon Lee:First author, contributed with design, execution, data collection, data analysis, interpretation of data and writing of the paper. Yong‐In Jo:Data collection and acquisition, drafting and reviewing. Sung‐Hee Yang:Corresponding author, contributed with design, execution, data analysis and reviewing. All Authors contributed with editing and approving the submitted manuscript.

## CONFLICT OF INTEREST

The authors declare no conflict of interest.
